# EGFR/BRAF/MEK co-inhibition for EGFR-mutated lung adenocarcinoma patients with an acquired BRAF^V600E^ mutation: a case report and review of literature

**DOI:** 10.20517/cdr.2021.98

**Published:** 2021-12-01

**Authors:** Ran Zeng, Lifeng Luo, Xianwen Sun, Zhiyao Bao, Wei Du, Ranran Dai, Wei Tang, Beili Gao, Yi Xiang

**Affiliations:** ^1^Department of Respiratory and Critical Care Medicine, Ruijin Hospital, Shanghai Jiao Tong University School of Medicine, Shanghai 200025, China.; ^2^Institute of Respiratory Diseases, Shanghai Jiao Tong University School of Medicine, Shanghai 200025, China.; ^3^Shanghai Key Laboratory of Emergency Prevention, Diagnosis and Treatment of Respiratory Infectious Diseases, Shanghai 200025, China.; ^4^Department of respiratory disease, Kashgar Prefecture Second People’s Hospital, Kashi 844000, Xinjiang, China.

**Keywords:** NSCLC, targeted therapy, resistance mechanisms, EGFR mutation, BRAF^V600E^ mutation, case report

## Abstract

Despite the promising initial anti-tumor efficacy of epidermal growth factor receptor-tyrosine kinase inhibitors (EGFR-TKIs), most advanced non-small-cell lung cancers (NSCLCs) progress eventually due to therapeutic resistance. V-Raf murine sarcoma viral oncogene homolog B1 (BRAF)^V600E^ mutation has been considered as an uncommon mutation that contributes to acquired resistance for EGFR-TKIs. In the presented case, BRAF^V600E^ mutation was detected as an acquired resistance-mediated mutation in a patient treated with osimertinib (a third-generation EGFR-TKI). The presented patient achieved partial regression and ongoing PFS of four months after the co-inhibition of osimertinib plus dabrafenib (BRAF inhibitor) and trametinib (MEK inhibitor). Our case further enriches the clinical evidence of the efficacy of EGFR/BRAF/MEK co-inhibition in patients with an acquired BRAF^V600E^ mutation, consistent with the review of the literature (eight cases). Additionally, our case highlights the important role of sample type, method, and platform of gene detection in patient management, life quality, and prognosis, as well as the understanding of acquired resistance mechanism.

## INTRODUCTION

Lung cancer is the leading cause of cancer-related mortality worldwide, and non-small-cell lung cancer (NSCLC) represents the histological subtype of 85% of lung cancer^[1]^. Great progress has been made in targeting the driver gene mutation for lung adenocarcinoma, a common subtype of NSCLC. Epidermal growth factor receptor (EGFR) mutations occur in 16% of advanced adenocarcinoma Caucasian patients, and the mutation frequency is as high as 61.1% in Asian females^[2,3]^. EGFR-tyrosine kinase inhibitors (TKIs) are the most common targeted therapy available for lung adenocarcinoma, including the first-generation inhibitors erlotinib, gefitinib, and icotinib; the second-generation inhibitor afatinib; and the third-generation inhibition osimertinib^[4]^. Although these therapies show promising initial anti-tumor activities, most advanced NSCLC cases eventually progress due to therapeutic resistance, also known as acquired resistance (AR). The alterations leading to EGFR-TKI resistance are divided into “on-target” resistance (alterations in targeted oncogene, EGFR) and “off-target” resistance (alterations in other downstream and parallel pathways)^[5]^. For alterations in downstream pathways, MAPK pathway reactivation plays an important role in the AR mechanism of EGFR-mutated lung cancer, including V-Raf murine sarcoma viral oncogene homolog B1 (BRAF)^V600E^ mutation^[6]^.

BRAF mutations are targeting oncogenic drivers, occurring in 3%-8% of lung adenocarcinomas. The common BRAF mutations consists of BRAF^V600E ^(50%), BRAF^G467A/V ^(35%), and BRAF^D549G ^(6%) mutations^[7,8]^. BRAF^V600E^ mutation induces constitutive BRAF activation in its monomeric form, activating downstream MEK-extracellular regulated protein kinases signaling^[9]^.

BRAF^V600E^ is also considered an uncommon mutation which contributes to AR in approximately 3% of patients receiving second-line osimertinib^[10,11]^. Although dabrafenib and trametinib have been approved for first-line treatment of metastatic NSCLC patients harboring BRAF^V600E^ mutation, and a pre-clinical study and several case reports have revealed the anti-tumor potential of co-inhibition treatment for post-line treatment after targeted therapy resistance, the efficacy and safety of EGFR/BRAF/MEK co-inhibition for EGFR-mutated NSCLC patients with an acquired BRAF^V600E^ mutation remain to be confirmed^[12-19]^.

Therefore, we report the therapeutic outcome of co-inhibition of EGFR, BRAF, and MEK for a lung adenocarcinoma patient who developed resistance to osimertinib. We also present a literature review of the clinical outcome and safety for EGFR/BRAF/MEK co-inhibition therapy in EGFR-TKI-treated NSCLC patients with acquired BRAF^V600E^ mutation, in order to further enrich the clinical evidence.

## CASE REPORT

**Figure 1:**
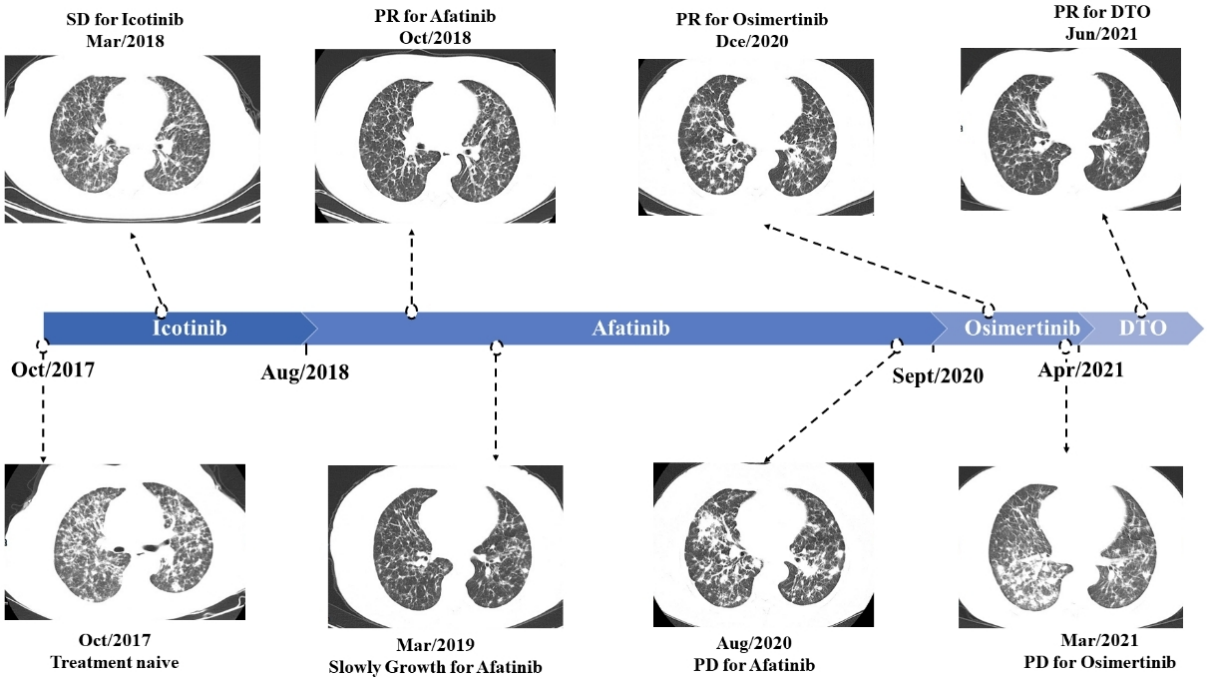
Radiologic images and timeline of the patient’s clinical course.

**Figure 2:**
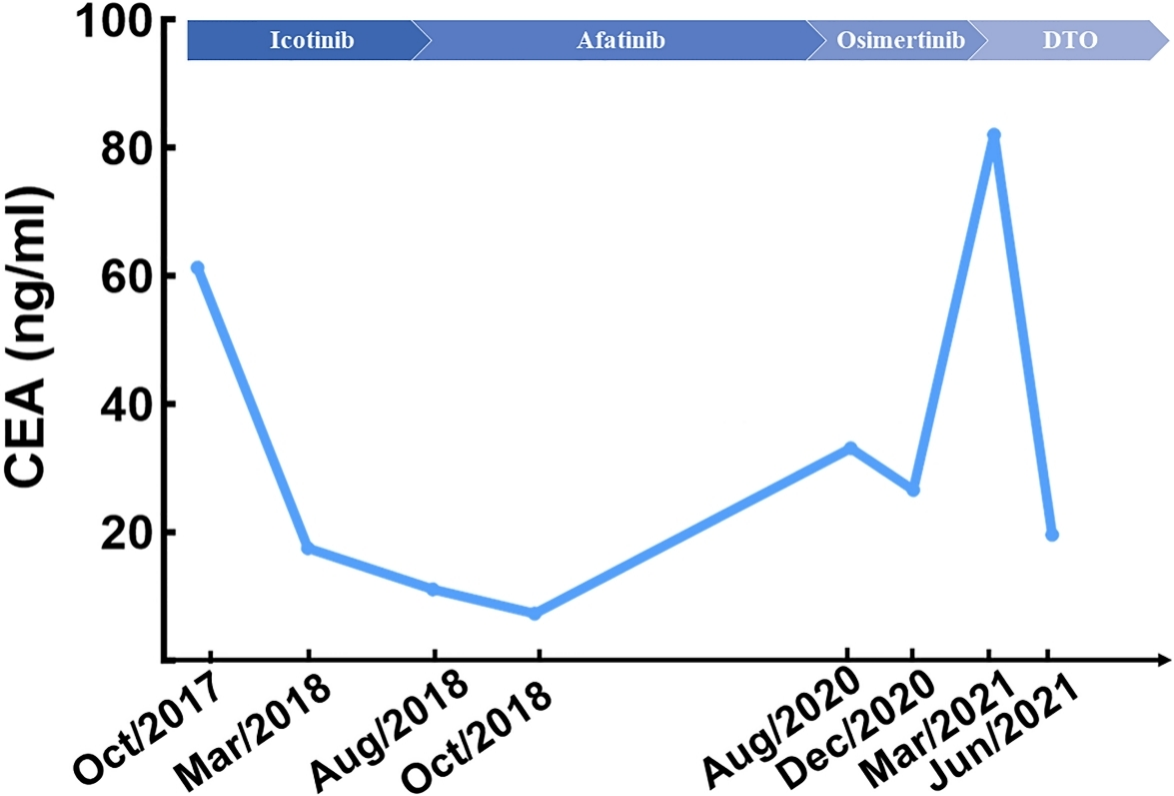
The changes in the carcinoembryonic antigen (CEA) level during the patient’s clinical course.

A 64-year-old female was admitted to the Department of Respiratory and Critical Care Medicine, Shanghai Ruijin Hospital in October 2017 due to the symptoms of cough and sputum. Further hospital examinations found scattered nodules in bilateral lungs, multiple enlarged (right supraclavicular, bilateral axillary, and bilateral inguinal) lymph nodes, and increased carcinoembryonic antigen (CEA) levels. Moreover, lung adenocarcinoma cells were found in biopsies of the lung nodules and right supraclavicular lymph node. After subsequent evaluation, she was diagnosed with lung adenocarcinoma, with the stage cT4N3M1a (tumor nodules in different ipsilateral lobes, supraclavicular node, and contralateral lobes), stage IVA (AJCC 8th edition), and lymphangitis carcinoma of the lung. Additionally, an EGFR exon 19 deletion (E19del) was identified by droplet digital polymerase chain reaction on lymph node biopsy. The whole course of treatment and changes of tumor indicators during the treatment are shown in Figures 1 and 2.

**Figure 3:**
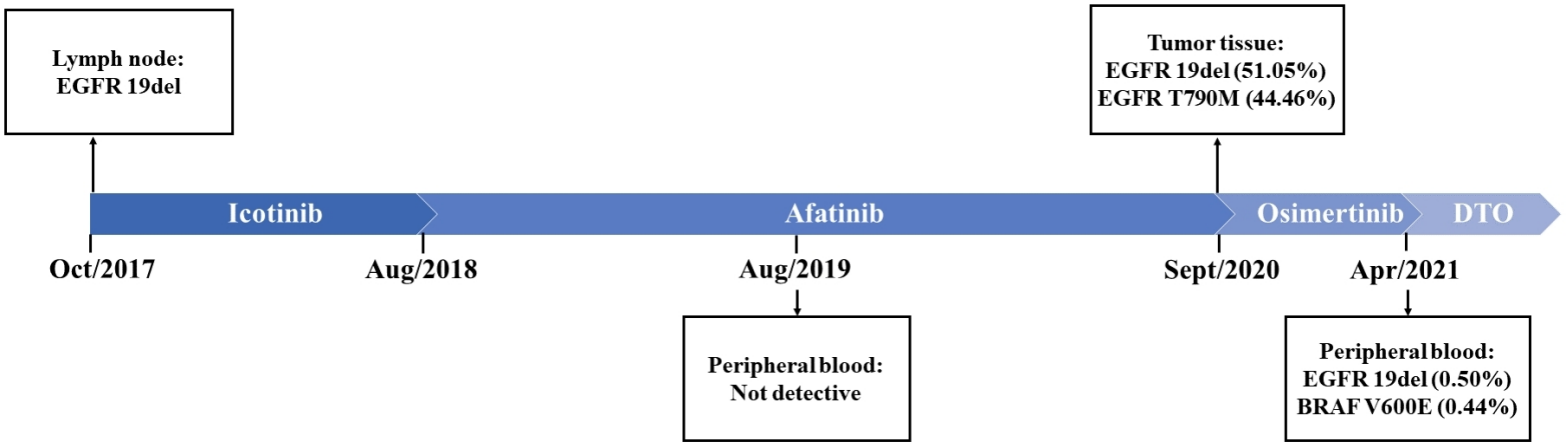
The mutations detected at different timepoints available from tissue or liquid biopsies.

Thus, she had clear indications for the first-generation EGFR-TKIs. Considering her abnormal liver function before hospitalization, we gave her icotinib (BETTA pharmaceutical, China) at a dose of 125 mg t.i.d, which has less hepatotoxicity than erlotinib and gefitinib. The treatment quickly prompted a stable disease based on criteria in Response Evaluation Criteria in Solid Tumors (RECIST) 1.1 and decreased CEA level. Five months later, she was diagnosed with drug-induced abnormal liver function (Grade 3) due to increased glutamine transaminase (ALT), aspartate amino transferase (AST), and bilirubin. Although icotinib was discontinued for one month and symptomatic treatment was given, abnormal liver function appeared again after the rechallenge of icotinib. Thus, afatinib (40 mg q.d) was given in August 2018. The chest computerized tomography (CT) after two months of afatinib showed partial response (PR) based on criteria in RECIST 1.1, with signiﬁcant regression of scattered nodules in bilateral lungs. However, in January 2019, her disease progressed slowly after a progression-free survival (PFS) of 17 months. Then, a secondary genetic testing was recommended, and she agreed to take a 168-gene panel (Burning Rock Biotech, Guangzhou, China) to test the driver mutation in her peripheral blood in April 2019. The results show that there was no targetable mutation, including E19del, EGFR-T790M, and BRAF^V600E ^[Figure 3].

Despise of our strong recommendation of secondary tissue biopsy, she refused any invasive manipulation and therapeutic regimen adjustment. After the continuation of afatinib until August 2020, the patient was admitted to our hospital due to chest distress, which could not be alleviated by rest. Further examination in August 2020 revealed the significant progression in pulmonary scattered nodules and a newly emerging enhanced brain nodule, combined with severe lymphangitis carcinoma and an increased CEA level. What is worse, she became increasingly dyspneic, along with presenting severe hypoxemia. At this time, she agreed to take ultrasound-guided percutaneous cutting needle biopsy, and E19del combined with EGFR-T790M mutation was identified by next-generation sequencing (NGS) targeting 168 genes [Figure 3]. Subsequently, the patient was treated with osimertinib (80 mg q.d) and achieved a PR in December 2020. Notably, brain MRI scanning showed the enhanced brain nodule was absorptive.

Unfortunately, in March 2021, she experienced disease progression again, along with highly increasing CEA levels and deep chest tightness. The chest scans showed enlargement of lung nodules, especially in the right lower lobe. NGS was performed by the patient’s plasma sample using a 168-gene panel and identified EGFR 19Del and BRAF V600E, but no EGFR T790M was detected [Figure 3]. Therefore, she began to receive osimertinib (80 mg q.d), dabrafenib (150 mg b.i.d), and trametinib (2 mg q.d) in April 2021. Her symptoms were significantly improved, and her CEA level decreased significantly within three weeks. The CT scan also demonstrated regressed lung nodules. All treatment-related adverse events, including rash, decreased appetite, fatigue, and repeated fever (range from 37.5 to 39.0 °C) were tolerable, graded as 1-2 (Common Terminology Criteria for Adverse Events version 5.0, CTCAE v5.0)^[20]^. The itching rash was distributed mostly in hands and feet, with spontaneous remission. Pyrexia occurred on the sixth day after the co-inhibition therapy. The co-inhibition therapy was discontinued for only two days when the temperature was as high as 39.0 °C. With physical cooling methods and usage of non-steroidal anti-inflammatory drug, pyrexia could be relieved. Notably, the laboratory test, including blood routine examination, etiological examinations, and C-reactive protein, showed no abnormality during her fever. At the last follow-up (September 2021), her disease evaluation remained stable.

## DISCUSSION

BRAF^V600E^ mutation is a rare oncogenic driver, occurring in 1.5%-4.0% of lung adenocarcinomas^[7]^. In a retrospective multicenter study enrolling 65 NSCLC patients harboring BRAF mutation, 54 patients harbored BRAF^V600E^ mutation and 11 had non-V600E mutations, including K601E, G469S, G469V, G469A, G596R, G466R, and T599dup^[21]^. An international, multicenter, noncomparative, and open-label trial (BRF113928) showed that the overall response rate of dabrafenib plus trametinib was 63% with response durations ≥ 6 months in 64% of responders^[22]^. Based on these results, in 2017, the Food and Drug Administration approved dabrafenib and trametinib for first-line treatment of metastatic NSCLC patients harboring BRAF^V600E^ mutation. The update of a phase 2 study (NCT01336634) showed promising efficacy of dabrafenib plus trametinib as second-line treatment, with a median PFS of 10.2 months and a median OS of 18.2 months^[23]^. However, no clinical trial has been conducted to confirm the efficacy of EGFR/BRAF/MEK co-inhibition in patients harboring E19 and BRAF^V600E^ after the therapy of osimertinib.

To our knowledge, the clinical outcome of EGFR/BRAF/MEK co-targeted therapy has been reported for eight cases. We summarize the clinical characteristics, therapeutic outcome, and toxicities in Table 1. Including our own case, ORR was 55.5% with PFS ≥ 6 months in 66.7% of these nine patients. Eight patients (88.9%) experienced adverse effects (AE), including pyrexia (5/9), nausea (2/9), paronychia (2/9), rash (2/9), fatigue (2/9), decreased appetite (2/9), vomiting (1/9), diarrhea (1/9), dysgeusia (1/9), pneumonitis (1/9), and increased creatine kinase (1/9). Most of these AE were evaluated as low grade, and only a few of them led to dose adjustment and co-inhibition therapy discontinuation. The study characterized the incidence, patterns, and management of pyrexia in patients receiving dabrafenib plus trametinib in clinical trials, concluding that pyrexia is the most common adverse event (61.3%) associated with dabrafenib plus trametinib. The incidence of pyrexia was highest early in treatment, decreased with time on treatment, and was manageable with dose interruption^[24]^. Pyrexia in the case occurred early in treatment, which was not associated with infectious inflammation. As shown in Table 1, patients (55.6%) with pyrexia tended to have a longer PFS (mean PFS: 8.4 months, 4.7-13.4 months) compared to patients without pyrexia (mean PFS: 4.2 months, 1.5-7.4 months). Although the association between pyrexia and clinical outcome is still unclear, there was a trend towards prolonged PFS in patients with pyrexia in our case and reported cases. Undoubtedly, the association between pyrexia and clinical outcome should be explored using larger sample sizes in future prospective clinical trials.

**Table 1:** Overview of literature for osimertinib-induced BRAF^V600^ mutation with the reported efficacy and treatment toxicities

**Author**	**Cases**	**Baseline EGFR mutation**	**Previous treatment**	**Mutaion profile at resistance to osimertinib**	**Treatment**	**Initial dose**	**Dose adjustment**	**Best overall response**	**Progression free time**	**Adverse effect (Grade)**
**Huang *et al*.^[13]^**	65 Male	EGFR 19del	Gefitinib → osimertinib	EGFR 19del/T790M, BRAF V600E	D+T+O^†^	D: 150 mg bid T: 1 mg qd O: 80 mg qd	Not need	SD	> 7.4 months^‡^	Diarrhea (G1), aronychia (G1)
**Solassol *et al*.^[15]^**	68 Female	EGFR 19del	Chemo → Afatinib → chemo → ICI → osimertinib → chemo + anti-VEGF	EGFR 19del/T790M, BRAF V600E	D+T/O^§^	D: 150 mg bid T: 2 mg qd O: 80 mg qd	Not need	SD	6 months	NR
**Ding *et al*.^[19]^**	63 Male	EGFR 19del	Gefitinib → osimertinib	EGFR 19del/T790M, BRAF V600E	D+T+O	D: 150 mg bid T: 2 mg qd O: 80 mg qd	Not need	SD	9 months	Pyrexia (G1-2) Aronychia (G1-2)
**Zhou *et al*.^[16]^**	69 Male	EGFR L858R	Post-operative recurrence, gefitinib→ chemo → osimertinib → chemo	EGFR L858R/T790M, BRAF V600E	D+T+O	D: 150 mg bid T: 2 mg qd O: 80 mg qd	Not need	PR	> 2 months	Rash (G2), decreased appetite (G2)
**Meng *et al*.^[17]^**	P1: 56 Female P2: 66 Male	P1: EGFR 19del P2: EGFR 19del	P1: Gefitinib → osimertinib P2: Afatinib → osimertinib	P1: EGFR E19del, BRAF V600E P2: EGFR 19del/T790M, BRAF V600E	P1: D+T+OP2: D+T+O	P1/P2: D: 150 mg bid T: 2 mg qd O: 80 mg qd	P1: discontiuation P2: D: 50 mg bid T: 0.5 mg qd O: 80 mg qd	/ PR	P1: 6 weeks P2: 13.4 months	P1: Pneumonitis P2: Pyrexia (G2), nausea,vomiting
**Ribeiro *et al*.^[18]^**	50 Male	EGFR 19del	Erlotinib → osimertinib + SBRT → chemo + ICI → chemo	EGFR 19del/T790M, BRAF V600E, PIK3CA mutation	D+T+O	D: 75 mg bid T: 1 mg qd O: 80 mg qd	D: 150 mg bid T: 2 mg qd O: 80 mg qd (Not succeed)	PR	8 months	Pyrexia, dysgueusia, nausea (G1) Fatigue (G1 → G2)
**Mauclet *et al*.^[14]^**	60 Female	EGFR 19del	Chemo + WBRT → ICI → erlotinib → osimertinib	EGFR E19del/T790M, BRAF V600E	D+T+O	D: 150 mg bid T: 2 mg qd O: 80 mg qd	D: 75 mg bid T: 1 mg qd O: 40 mg qd	PR	7 months	Increased creatine kinase (G3) Prexia (G2)
**This paper**		EGFR 19del	Icotinib → afatinib → osimertinib	EFGR 19del, BRAF V600E	D+T+O	D: 150 mg bid T: 2 mg qd O: 80 mg qd	Not need	PR	> 4.7 months	Pyrexia (G2), rash (G1), fatigue (G1), nausea (G1), decreased appetite (G1)

D + T + O: dabrafenib and trametinib plus osimertinib; ^‡^>: treatment ongoing; ^§^D/T and O treatments were alternated every month. Chemo: Chemotherapy; ICI: immune checkpoint inhibitor; PR: partial response; SD: stable disease; PD: progressive disease; SBRT: stereotactic body radiotherapy; WBRT: whole brain radiotherapy; NR: not reported.

Immunotherapy and chemotherapy are suggested options after the failure of EGFR-TKI treatment according to the National Comprehensive Cancer Network Guidelines for Non-Small Cell Lung Cancer (2021). However, for BRAF^V600E^ mutated patients, a previous study showed limited efficacy for chemotherapy, with a first-line objective response rate (ORR) of 23% and second-line ORR of only 9%^[25]^. As for immunotherapy, research has revealed that ORR with immune checkpoint inhibitors (ICIs) was 24% in BRAF mutation patients, although BRAF mutation NSCLC was associated with high levels of PD-L1 expression^[26]^. In JTO Clinical and Research Reports, Zhang *et al*.^[27]^ reported that BRAF^V600E^ mutation was associated with worse clinical outcome for ICIs (median OS, BRAF^V600E ^*vs*. non-V600E: 5 months *vs*. 14 months, *P *= 0.017). Compared with chemotherapy and immunotherapy, better clinical outcomes have been observed in patients treated by anti-BRAF with or without anti-MEK therapy^[21,28]^. Taken together, it is worth considering EGFR/BRAF/MEK co-inhibition as second-line treatment options for EGFR-mutated NSCLC patients with BRAF^V600E^ mutation.

With the development of genetic tests, great advances have been made in targeted therapy for lung cancer. The accurate identification of predictive genetic alterations is important for patient management, life quality, and prognosis, as well as the understanding of AR mechanism to different therapies^[4]^. The case is a typical long-term treatment management guided by multiple genetic tests. At the time when cancer cells of our patient developed afatinib resistance, an EGFR T790M was identified by NGS on lung biopsy but without targeting driver mutation found in her peripheral blood NGS test. This result might be due to the higher sensitivity in the genetic test derived from tumor tissues. However, she suffered a rapid disease progression with a very short duration of response. It is reported that, despite the high sensitivity, tissue genetic analysis has difficulty representing the whole driver gene mutation profile because of spatial heterogeneity. The reason for the fast progression for osimertinib in our case might be the existence of other undetected resistance mechanisms. The principal manifestation of this patient’s progressive disease was lymphangitis carcinoma of the lung, which increased the difficulty of tumor tissue biopsy. Liquid biopsy may be better than tissue analysis, as it has the potential to represent tumor heterogeneity and clonal diversification^[29]^. Several studies emphasized the guiding role of monitoring the dramatic change of the driving mutation by circulating free DNA test^[30,31]^. Therefore, liquid (peripheral blood and pleural effusion) biopsy and tissue genetic analysis complement each other, and the reasonable choice of detection sample type, method, and platform plays an important role in the accuracy of precision medicine, especially in patients suffering drug resistance. Additionally, a study indicated that serum CEA determinations are a feasible, noninvasive option for monitoring and prognosis^[32]^. Similar to the previous result, in our case, the change of serum CEA also remained consistent with anti-tumor response.

In conclusion, a strong and fast response to osimertinib plus dabrafenib and trametinib was observed in this case of resistance in a patient with BRAF^V600E^ mediated AR under osimertinib therapy, which has lasted for more than four months. Our case further enriches the clinical evidence of the efficacy of EGFR/BRAF/MEK co-inhibition in patients with an acquired BRAF^V600E^ mutation, consistent with the review of the literature (eight cases). Additionally, our case highlights the important role of sample type, method, and platform of gene detection in patient management, life quality, and prognosis, as well as the understanding of acquired resistance mechanism.

## DECLARATIONS

### Copyright

© The Author(s) 2021.
